# Reconstruction of Axonal Projections of Single Neurons Using PointTree

**DOI:** 10.21769/BioProtoc.5616

**Published:** 2026-03-05

**Authors:** Lin Cai, Xuzhong Qu, Junwei Wang, Yuan Shen, Tingwei Quan

**Affiliations:** 1 Britton Chance Center for Biomedical Photonics, Wuhan National Laboratory for Optoelectronics, Huazhong University of Science and Technology, Wuhan, Hubei, China; 2 MOE Key Laboratory for Biomedical Photonics, Wuhan National Laboratory for Optoelectronics, Huazhong University of Science and Technology, Wuhan, Hubei, China

**Keywords:** Neuron reconstruction, Axonal projections, Points assignments, Dense reconstruction, Brain circuit mapping

## Abstract

The morphology of single-neuron axonal projections is critical for deciphering neural circuitry and information flow in the brain. Yet, manually reconstructing these complex, long-range projections from high-throughput whole-brain imaging data remains an exceptionally labor-intensive and time-consuming task. Here, we developed a points assignment-based method for axonal reconstruction, named PointTree. PointTree enables the precise identification of the individual axons from densely packed axonal population using a minimal information flow tree model to suppress the snowball effect of reconstruction errors. In this protocol, we have elaborated on how to configure the required environment for PointTree software, prepare suitable data for it, and run the software. This protocol can assist neuroscience researchers in more easily and rapidly obtaining the reconstruction results of neuronal axons.

Key features

• Optimized for mapping long-range axons that connect distant brain regions in dense or crossover scenarios.

• Enables high-fidelity (F1-score > 80%) reconstruction of hundreds of GB of large-volume imaging data.

• Compatible with LSM, fMOST, and HD-fMOST systems for diverse neuroimaging datasets.

## Graphical overview


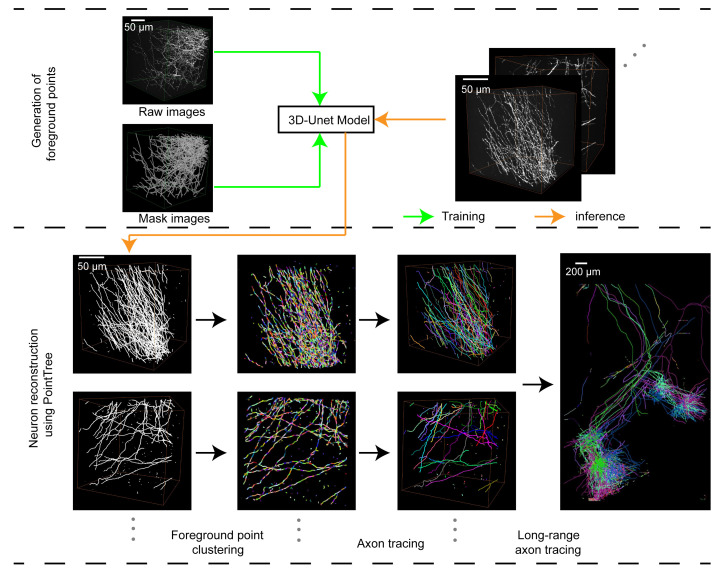


**Workflow for reconstruction of axonal projections using PointTree**

## Background

Neuronal axonal projections serve as a crucial indicator for identifying neuron types, constructing neuronal circuits, and gaining deeper insights into how information is transmitted in the brain [1,2]. Advancements in optical imaging and molecular labeling techniques have enabled us to observe the entire mouse brain with single-axon resolution, thereby providing a valuable database for studying neuronal projection patterns [3–6]. However, reconstructing long-range projecting axons demands extensive manual work on massive volumetric images, creating a major obstacle for high-throughput neuronal projection mapping [7]. The key problem is the lack of precise algorithms to avoid errors, as current methods struggle with densely packed axons, mainly focusing on skeleton point localization and connection [8]. During the process of connecting skeleton points, reliance is solely placed on limited information, such as the spatial positions of the skeleton points and the neighboring image signals of each skeleton point, which usually include signals from other axons.

To overcome this problem, we propose a new method based on point assignment named PointTree [9]. In the PointTree framework, the foreground point set is divided into a series of interconnected ellipsoidal regions. Subsequently, the connection relationships among the center points of these partitioned point sets are established by leveraging the directional characteristics of the ellipsoidal regions, their regional connectivity, and positional information, thereby enabling axon tracing. We have designed specific constraints for partitioning the foreground point set to ensure that each divided ellipsoidal region is localized within a single axon. Additionally, the shape of these ellipsoidal regions is utilized to facilitate connections between the centers of the partitioned foreground point sets. These features effectively enable the separation of densely packed neurites. Furthermore, we designed a minimal information flow tree model to suppress the cumulative reconstruction error. By employing PointTree, we have successfully reconstructed long-range neuronal projections from image blocks sized in the hundreds of gigabytes, achieving a reconstruction accuracy exceeding 80%.

Currently, the PointTree software only works on foreground point sets. It requires the radius of the cylinder-shaped structures made up of these foreground points to not be more than 2 voxels. Because of this, getting the foreground point sets accurately is really important. While the ellipsoid extraction in PointTree effectively localizes neuronal centerlines, it may occasionally produce split fibers in regions with thick signal intensity. The Seg_Net refinement reduces such duplications but may also suppress legitimate weak signals, leading to discontinuities in sparse or low-contrast regions. Users working with densely labeled or high-noise data should be aware of this inherent trade-off between split-reduction and sensitivity to faint structures. Second, there are still mistakes in the PointTree reconstruction process, so we need to introduce how to correct these mistakes. Finally, we should clearly point out the specific situations where the PointTree software can be used. Based on these reasons, we have made and shared this corresponding protocol (a set of operating steps) to explain in a clear, step-by-step way how the PointTree software can get accurate neuron reconstruction results from neuron images.

## Software and datasets

1. Dataset for PointTree, https://zenodo.org/records/15589145 , MIT license, free

2. PointTree, https://zenodo.org/records/15589145 , MIT license, free

3. GTree, https://github.com/GTreeSoftware/GTree , Revised MIT license, free

4. Seg_Net, https://github.com/FateUBW0227/Seg_Net , MIT license, free

**Table 1.** Dataset for PointTree content

Name	Content
Data_for_different_SNR.zip	Data blocks with different SNRs (signal-to-noise ratio) from fluorescence micro-optical sectioning tomography (fMOST), light sheet microscopy (LSM), and high-definition fluorescence micro-optical sectioning tomography (Hd-fMOST).
Data_of_difficult_structure.zip	Data blocks with difficult structures (crossover and closely parallel fibers).
Dataset_for_training.zip	Dataset for training of Seg_Net.
Full_data1.zip	A set of brain data (voxel size: 11,226 × 8,791 × 1,486, including all intermediate results).
Full_data2.zip	Another set of brain data (voxel size: 10,739 × 11,226 × 3,921, including all intermediate results).
Merge_process.zip	Dataset for validating merge process.
MIFT_data.zip	Dataset for validating minimal information flow tree (MIFT).
Patch_data.zip	Dataset for comparison with other methods.
PointTree_software.zip	PointTree software.
ReadMe.MD	A brief description of the content of the Dataset for PointTree.
UserGuider.pdf	A user guider for PointTree.

Users can use Full_data1.zip and Full_data2.zip to validate this protocol. Others are datasets for validating the algorithm.

**System requirements**

To improve reproducibility, the following hardware specifications are recommended for the computational setup.

1. Operating system: Windows 11.

2. CPU: A multi-core CPU (central processing unit) is required. A minimum of 4 cores at 3.5 GHz base clock speed is necessary, but a configuration of 8 or more cores at 4.0 GHz or higher is recommended for optimal performance.

3. Memory: 16 GB RAM (random access memory, minimum), 32 GB RAM (recommended).

4. Storage: Sufficient space for storing image data as well as the processed segmentation and the tracking results.

5. Graphics: Nvidia GPU (graphics processing unit) with CUDA (compute unified device architecture) support (VRAM, video RAM exceeding 8 GB, CUDA 11.1-compatible) is required to run PointTree. PointTree has been successfully tested on Nvidia GeForce GTX 1080Ti and GTX 1060 graphics cards. We recommend using the latest GPU drivers to ensure compatibility and optimal performance.

**Video 1.**
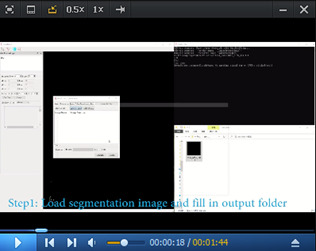
Video demonstration of PointTree software

## Procedure

Input data description:

Image resolution: 0.35 × 0.35 × 1.0 μm.

Voxel size: 10,000 × 10,000 × 2,000. (We have tested on datasets with voxel size of 10,739 × 11,226 × 3,921 and 11,226 × 8,791 × 1,486.)

SNR: >2. (We have tested datasets with SNR ranging from 2 to 14.)

File format: Tiff

Bit depth: 8-bit or 16-bit.

Data chunking: The full image volume is partitioned into overlapping cubes of size 512 × 512 × 512 voxels, with an overlap of 25 voxels between adjacent cubes.

**A. Software installation**

1. Neuron reconstruction tools installation

PointTree is a software tool designed to reconstruct long-range neuronal projections. Currently, it is only compatible with Windows operating systems. To launch the software, decompress the downloaded package and double-click PointTree.exe.

GTree is a fast, cross-platform (Windows, Linux) software designed for neuroscience research, offering free visualization and analysis of neuronal imaging data [10]. GTree enables reconstruction of neuronal populations from single images or terabyte-scale whole-brain image stacks. As an integrated tool, it supports soma localization, automated/manual neuron tracing, neurite morphology reconstruction, and manual editing functions. The installation procedure of GTree software can be found at https://github.com/GTreeSoftware/GTree/wiki .

GTree is not an alternative to PointTree. Instead, they serve distinct and sequential roles in the workflow. PointTree is the core analysis software for reconstructing long-range axons from pre-segmented images. All users must install PointTree to perform the primary analysis described in this protocol. GTree is an optional annotation tool designed specifically for generating high-quality training data. Its purpose is to help users manually label raw neuronal images when they wish to train a custom segmentation model (Seg_Net) but lack annotated data. The output of GTree (annotation files) is used to train Seg_Net, which in turn produces the segmentation results required by PointTree.

2. Seg_Net installation

a. Install Python dependencies: The code has been successfully tested with Python 3.9. To ensure maximum stability, we recommend using Python 3.9. Download the repository using:

git clone https://github.com/FateUBW0227/Seg_Net.git

It is recommended to use a virtual environment to manage the packages (Optional):

python -m venv .venv

.venv\Scripts\activate

Ensure the package management tool pip is up to date. The main dependencies (e.g., numpy==1.24.3, tifffile==2024.5.3, h5py==3.8.0, scikit-image==0.20.0, etc.) are listed in the requirements.txt file. Install them all with one command:

pip install -r requirements.txt

There may be issues with the Python version not being compatible with the installed libraries during the dependency installation process. The version constraints of the dependent packages can be appropriately relaxed.

3. Install the pytorch-3dunet package

Pytorch-3dunet provides a PyTorch-based UNet3D model for subsequent neural image segmentation tasks [11]. A pytorch-3dunet-master.zip file is provided in the Seg_Net repository. Users can extract it directly in the working directory and install the package using the following commands:

cd pytorch-3dunet-master

pip install -e .

**B. Generation of foreground points from neuronal images**

**Figure 1.**
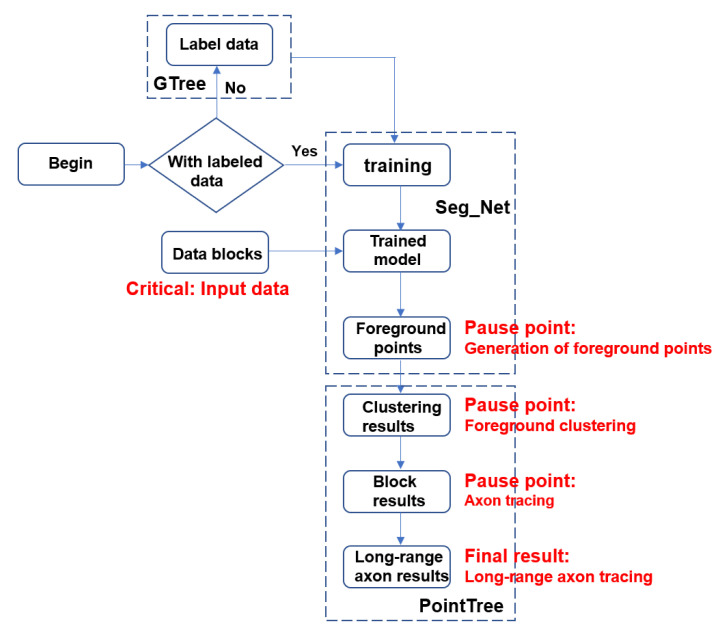
Workflow of long-range axon tracing via Seg_Net and PointTree

All procedures, demonstration data, and expected operational results related to the following workflows (Figure 1) can be referenced from the GitHub repository (Seg_Net) and Zenodo data links provided in the Software and datasets section.

1. Preparing datasets for training Seg_Net

Before initiating the training of the Seg_Net, it is essential to prepare 3D neuronal images along with their corresponding distance field maps to serve as training labels. The training dataset should be representative of the biological variance. As an example, we selected 20 data blocks of size 512 × 512 × 512 voxels, comprising 10 blocks with densely distributed axons and 10 blocks with sparsely distributed axons from distant regions. The process of generating these training labels entails manually annotating neuronal skeletons using the semi-automatic software GTree. Subsequently, 3D distance field maps are derived by interpolating the skeleton points and calculating their distance fields. These maps act as ground truth for supervising the output of the segmentation network, guaranteeing that the segmented neuronal processes maintain a relatively consistent radius. For further details on the calculations, please refer to Seg_Net repository.We provide a training set named dataset_ for_training.zip. After unzipping, you will see three folders:

Raw_images: Raw input images stored in .tif format, serving as the unprocessed data source for model input.

Mask images: Ground truth masks for training, typically generated through manual annotation or based on SWC files.

swc_files: Standard neuronal morphology files stored in .swc format, recording the skeleton model and topological connectivity of neurons.

We can also generate mask images from labeled SWC files using the PreMakeData.py program, converting SWC files describing neuronal morphology into 3D distance field maps and saving them as .tif image files. PreMakeData.py can be downloaded in Seg_Net repository.

2. Preprocessing the training dataset

In this work, the data was split into training (for model learning), validation (for model selection), and testing (for final evaluation) sets. For end-users, their own images represent a separate application dataset. To apply our method, we recommend that users annotate a subset of their data for training/validation and reserve the rest for evaluating the performance of their customized model.

Prior to training, the image stacks require preprocessing. To balance computational efficiency and information retention, the data is generally cropped into sub-volumes, each with a size of 128 × 128 × 128. This size mitigates the high GPU memory requirements associated with larger inputs while avoiding the loss of critical semantic information that can occur with smaller patches. In our case, each image stacks have the size of 512 × 512 × 512. These stacks are subdivided into 64 sub-volumes to form the training dataset.

Step1. Place the input images and mask images into the following folders:

./Test/raw_image/ (for storing raw images)

./Test/mask/ (for storing mask images)

Step2: Create the following directory structure:

“./Test/Save/train/image”

“./Test/Save/train/mask”

Step3. Execute the following command to synchronously crop the raw images and mask images:

cd ./preprocess

Cut_data.exe

A configuration file (config.txt) is provided alongside Cut_data.exe, containing the following customizable entries:

“Image_dir ./Test/raw_image/

Mask_dir ./Test/mask/

Save_dir ./Test/Save/”

Users may modify the paths after Image_dir and Mask_dir to point to their own dataset directories and set the Save_dir path to any preferred output location. Each path entry must conclude with a semicolon.

Once you have executed Cut_data.exe, kindly be patient and allow the program to carry out its processing tasks. When you notice the terminal continuously displaying the message “0 100 DONE,” it serves as an indicator that the image-cropping procedure has commenced. Continue waiting until this process is fully completed.

Simultaneously, the program will automatically generate two files: train.txt and test.txt. These files will contain the lists of filenames for the partitioned training and validation sets, respectively.

After cropping is complete, the file directory structure is organized as follows:

./

 └── Test/

 ├── raw_image/ # Storing input images

 ├── mask/ # Storing mask images

 └── Save/

 └── train/

 ├── image/ # the cropped raw images

 ├── mask/ # the cropped mask images

 ├── test.txt # List of filenames for the test se=t

 └── train.txt # List of filenames for the training set

3. Running the training program

Before executing the training script Train.py, set the training set folder path in Train.py to ./Seg_Net/preprocess/Test/Save/train. The program will automatically split the input images into training and validation sets according to the predefined text files. Training logs are recorded in the ./logs directory, while the optimal model weights, determined by minimal loss, are automatically checkpointed to the ./ModelSave/ folder.

4. Running the image prediction program

Prior to launching the BigImgPredict.py program, it is essential to properly configure three crucial paths. These include the input image path, denoted as imgPath, which specifies where the original images are located; the result saving path, denoted as savePath, where the output segmentation results will be stored; and the model weight path, denoted as modelPath, which points to the location of the model’s weight files.

Once we have set up these paths and run the program, the segmentation results will be saved as files in the .tif format. We can then utilize the GTree software to view these results in a three-dimensional context.

**C. Neuron reconstruction using PointTree**

1. Data description

The input data for the software is generated through segmentation using Seg_Net. As previously mentioned, the segmented images do not directly depict the foreground points of neuronal fibers. Instead, they consist of a series of cylindrical structures aligned along the central axes of the nerve fibers.

The software produces an SWC file that captures the morphological structure of neurons. This text-based file records the identification number, three-dimensional coordinates, and parent node information for each node within a tree-like structure, comprehensively representing the topological connectivity and geometric attributes of the neurons.

For demonstration purposes, we provide the Full_data dataset in Table 1, which includes four subsets of data within the folder. The “segment” folder contains the segmentation results, the “gauss” folder holds the clustered results, the “skeleton” folder includes the patch tracing results along with the starting points and batch selection files, and the “res” folder contains the long-range tracing results (https://zenodo.org/ records/15589145).

2. Foreground point clustering module

This module processes foreground points (i.e., neuronal fiber regions) obtained from segmentation network results. It employs a constrained Gaussian mixture model (GMM) to cluster these foreground points, generating a series of cylindrical regions, each representing a local segment of a nerve fiber. This step lays the foundation for subsequent tracing and requires CUDA 11.1-compatible GPU acceleration for efficient computation. The input data is foreground points generated by the segmentation network (e.g., volumetric data of size 512 × 512 × 512). The output files are saved in .swc format, containing cylindrical regions formed by clustered foreground points. Each cylindrical region represents a local segment of a neuron.

Within the PointTree software interface, proceed with the following step:

a. Click the second button in the upper-left corner to enter the local patch generation module.

b. Click *Open Folder* under *Save Directory* to select the output directory for results.

c. Click *Open Folder* under *Image Data* to select the directory containing segmentation results (e.g., .tif files from the segment folder in the provided dataset).

d. Click the *GPU* button to display available GPU devices. Select an appropriate GPU (ensure enough VRAM is reserved).

e. Click *Confirm* to start local patch generation. Upon completion, a “Patch generation done” message will appear.

**Figure 2.**
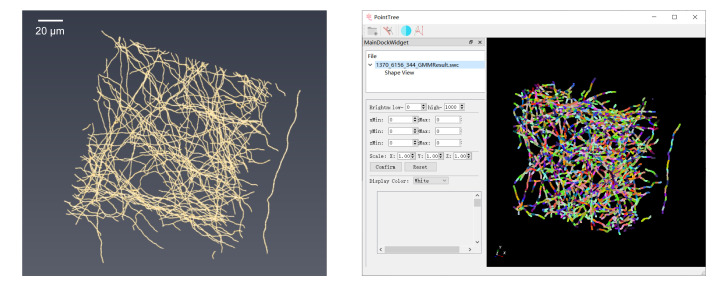
Display of foreground point clustering results. (Left) Foreground points in the neural image. (Right) Clustering performed using PointTree software, with different colors representing distinct clusters.

The generated .swc files can be dragged into the software and visualized in 3D by right-clicking in the *Shape View* window, as shown in Figure 2.

3. Axon tracing module

Based on the generated clustered local patches, this module employs a 0–1 assignment model to establish connections between different columnar regions, thereby forming a local neurite skeleton. The process utilizes the minimum volume ellipsoid to describe the geometric features of each columnar region and constructs continuous neurite paths by optimizing a connection cost function. The input data consists of clustered local patch files, and users may optionally download the .swc files under the “gauss” folder from the “Local Patch Generation” data. The output data is a local neurites skeleton file saved in .swc format.

Steps to operate:

a. Click the third button in the upper-left corner to enter the axon tracing module.

b. Click *Open Folder* under *Save Directory* to select the directory for saving results.

c. Click *Open Folder* under *Image Data* and select the clustered result .swc file.

d. Click *Trace* to initiate the local tracing process. Upon completion, the terminal will display a message box saying “Local Tracing Done.”

**Figure 3.**
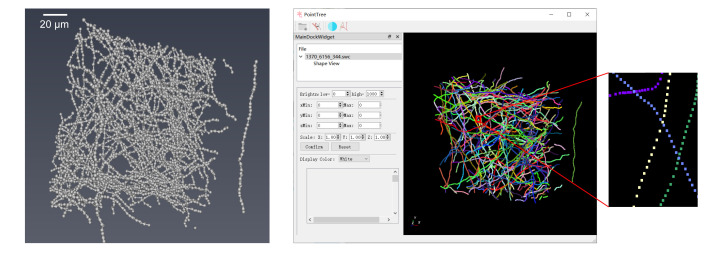
Presentation of axonal tracing outcomes. (Left) Clustered foreground points serving as the input for PointTree in axonal tracing. (Right) Traced axonals, with different colors indicating distinct individual axonals.

The generated results will be saved in .swc format under the “skeleton” folder and can be dragged into the software for 3D visualization by right-clicking in the *Shape View*, as shown in Figure 3.

4. Long-range axon tracing module

This module is used to reconstruct long-range axonal projections of individual neurons in the brain. Building upon the local tracing results, it introduces the Minimum Information Flow Tree (MIFT) model to correct the skeleton topology and suppress the accumulation of erroneous linkages. For the reconstruction results of adjacent image patches, it uses another 0–1 assignment model to merge them, ensuring the continuous reconstruction of the entire neuron. The module supports automatic tracing from a user-specified starting point (such as the soma location) to the distal axonal terminals.

The input data consists of the local neurites tracing results. These are multiple .swc files generated from the “Axon tracing” step, with each file containing the local neurites skeleton extracted from an image patch. The input folder includes three parts: 1) .swc files storing the axonal tracing results; 2) .txt file containing the names of all merged .swc files; and 3) a starting point location file (.txt format) specifying the spatial coordinates of the starting point for each neuron. The output is a complete neuron reconstruction file saved in .swc format.

Steps to operate:

a. Click the fourth button in the upper left corner to enter the long-range tracing mode.

b. Click *Open Folder* under *Save Directory* to select the folder for saving results.

c. Click *Open Folder* under *Image Data* and input the start point file (you may choose the sample file head.txt from the skeleton folder). The input for this step is similar to the previous one (the data file name represents the coordinates of the data blocks).

We import multiple skeleton files in bulk using a .txt list to simplify the process. This list file should contain the paths of all skeleton files (.swc) to be processed. Only the target reconstruction range needs to be covered; whole-brain data is not required. You may refer to the sample file list.txt in the skeleton directory for the specific format.

d. Click *Trace*; the long-range tracing module will begin.

**Figure 4.**
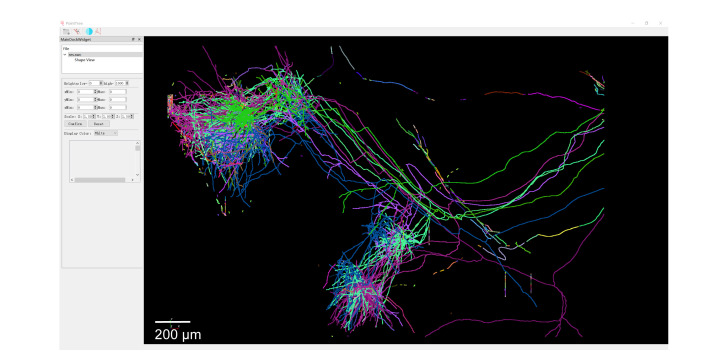
Display of long-range axon reconstruction

When the process is completed, the terminal will display “SAVE ‘save_path/Global_res/res.swc’ SAVE COST TIME Merge done,” and a message box saying “Long range tracing done” will appear to indicate completion. The generated results will be saved in the Global_res folder in res.swc format and can be dragged into the software for 3D visualization by right-clicking in *Shape View*, as shown in Figure 4.

**Result interpretation**

Output of Seg_Net: A TIFF file containing the binary segmentation mask, where white voxels represent the foreground signal. This file serves as the primary input for the PointTree software.

Output of PointTree: A swc file representing the final neuronal reconstruction. This text file defines the neuron as a tree graph, with each line detailing a node’s properties: ID, type, x, y, z, radius, and parent_ID. The reconstruction can be visualized within PointTree, where distinct colors are automatically assigned to differentiate individual neurons.

We provided the time consumption of each intermediate step for PointTree to reconstruct two different datasets in Table 2.

**Table 2.** Time cost of PointTree

**Number of blocks (each size: 512 × 512 × 512)**	**Points clustering (min)**	**Axon** **tracing (min)**	**Long-range** **axon tracing (min)**
254	23	18	3
821	22	35	3

GPU usage during point clustering is 6–10 GB.

## Validation of protocol

The data for Figures 2–4 is available at https://zenodo.org/records/15589145 . The raw image blocks are extremely large and can be made available on request from the corresponding author.

**Figure 5.**
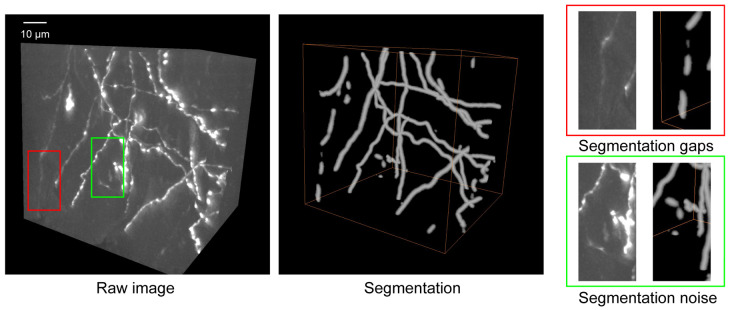
Some troubleshooting cases in segmentation. The raw image shows the original neuronal structures. Segmentation displays the result obtained using Seg_Net. Segmentation gaps (red box) highlight regions where Seg_Net failed to detect the complete neuron. Segmentation noise (green box) indicates areas where background artifacts were incorrectly classified as neurons. Scale bar: 10 µm.

This protocol has been used and validated in the following research article:

- Cai et al. [9]. Automatic and accurate reconstruction of long-range axonal projections of single-neuron in mouse brain. *eLife* (Figure 3, panel 1; Figure 4, panel 2; Figure 5, panel 1; Figure 6).

## General notes and troubleshooting

**Problem 1**: Users may encounter dependency conflicts during installation.

Possible cause: The requirements.txt file specifies a range of compatible versions, but these may conflict with existing packages or system dependencies in some Python environments.

Solution: We recommend using a virtual environment to isolate the project’s dependencies. If conflicts persist, the version constraints can be manually adjusted.

**Problem 2:** PointTree fails to launch, reporting missing msvcr.dll and msvcp.dll files.

Possible cause: This error indicates that the Microsoft Visual C++ 2013 Redistributable runtime libraries are required. (Note: This is a shared dependency with GTree.)

Solution: Download and install Microsoft Visual C++ 2013 Redistributable from the official Microsoft website.

**Problem 3:** Poor segmentation performance on a new dataset.

Possible cause: The provided dataset is from fMOST. Significant domain shift in image characteristics (e.g., resolution, SNR) between fMOST and the new dataset can lead to segmentation errors.

Solution: For optimal results on a substantially different dataset, we recommend training a new segmentation model using the new dataset. First, use GTree to manually annotate some data blocks from the new dataset, and then use the Seg_Net to train a new model with the annotated data blocks.

**Problem 4:** Unable to install dependency with Python 3.12 or later version.

Possible cause: The version in requirements.txt is not compatible with Python 3.12 or later.

Solution: The best way is to create a virtual environment and change the Python version to 3.9. If users insist on using Python 3.12, update requirements.txt file to specify numpy == 1.26.0, scikit-image == 0.26.0, h5py >= 3.8.0, and imagecodecs >= 2023.3.16.

**Problem 5:** Segmentation results contain gaps/discontinuities or noise.

Possible cause: The training dataset may be deficient in samples containing faint/weak signals (Figure 5).

Solution: Most noise components are spatially isolated from the true signal. PointTree incorporates an internal denoising module that automatically removes these isolated fiber fragments. Enhancing the training dataset with additional weak-signal samples can partially mitigate segmentation discontinuities. For short gaps, PointTree includes a built-in connection function to bridge the discontinuities. If gaps persist in the final reconstruction, manual correction is supported within the PointTree framework.
